# 

**Published:** 1902-04

**Authors:** 


### The East Texas Medico-Chirurgical Society.

This society will meet in Palestine May 29-30, prox. This will be an important meeting. Officers for ensuing year will be elected. A new constitution and by-laws will be adopted. The committee appointed to assist in enforcing the new medical law will report, and other important business matters will be attended to, besides the reading of interesting and instructive papers. (See May issue of "Red Back," the official organ, for program.)

It is hoped that every member will be present, and that every physician in East Texas who is not a member will become one at this meeting.

